# Feasibility of Velocity‐Selective Arterial Spin Labeling in Breast Cancer Patients for Noncontrast‐Enhanced Perfusion Imaging

**DOI:** 10.1002/jmri.27781

**Published:** 2021-06-13

**Authors:** Suzanne L. Franklin, Nora Voormolen, Isabell K. Bones, Tijmen Korteweg, Martin N. J. M. Wasser, Henrike G. Dankers, Daniele Cohen, Marijn van Stralen, Clemens Bos, Matthias J. P. van Osch

**Affiliations:** ^1^ C.J. Gorter Center for High Field MRI, Department of Radiology Leiden University Medical Center Leiden The Netherlands; ^2^ Center for Image Sciences University Medical Centre Utrecht Utrecht The Netherlands; ^3^ Leiden Institute for Brain and Cognition Leiden University Leiden The Netherlands; ^4^ Department of Radiology Leiden University Medical Center Leiden The Netherlands; ^5^ Department of Pathology Leiden University Medical Center Leiden The Netherlands

**Keywords:** breast cancer, arterial spin labeling, ASL, DCE‐MRI, noncontrast enhanced, screening

## Abstract

**Background:**

Dynamic contrast‐enhanced (DCE) MRI is the most sensitive method for detection of breast cancer. However, due to high costs and retention of intravenously injected gadolinium‐based contrast agent, screening with DCE‐MRI is only recommended for patients who are at high risk for developing breast cancer. Thus, a noncontrast‐enhanced alternative to DCE is desirable.

**Purpose:**

To investigate whether velocity selective arterial spin labeling (VS‐ASL) can be used to identify increased perfusion and vascularity within breast lesions compared to surrounding tissue.

**Study Type:**

Prospective.

**Population:**

Eight breast cancer patients.

**Field Strength/Sequence:**

A 3 T; VS‐ASL with multislice single‐shot gradient‐echo echo‐planar‐imaging readout.

**Assessment:**

VS‐ASL scans were independently assessed by three radiologists, with 3–25 years of experience in breast radiology. Scans were scored on lesion visibility and artifacts, based on a 3‐point Likert scale. A score of 1 corresponded to “lesions being distinguishable from background” (lesion visibility), and “no or few artifacts visible, artifacts can be distinguished from blood signal” (artifact score). A distinction was made between mass and nonmass lesions (based on BI‐RADS lexicon), as assessed in the standard clinical exam.

**Statistical Tests:**

Intra‐class correlation coefficient (ICC) for interobserver agreement.

**Results:**

The ICC was 0.77 for lesion visibility and 0.84 for the artifact score. Overall, mass lesions had a mean score of 1.27 on lesion visibility and 1.53 on the artifact score. Nonmass lesions had a mean score of 2.11 on lesion visibility and 2.11 on the artifact score.

**Data Conclusion:**

We have demonstrated the technical feasibility of bilateral whole‐breast perfusion imaging using VS‐ASL in breast cancer patients.

**Evidence Level:**

1

**Technical Efficacy:**

Stage 1

Breast MRI is the most sensitive tool for detection of breast cancer^1^ with a sensitivity at least twice as high as mammography.^1^ The specificity of breast MRI is increased by use of multiparametric (mpMRI) protocols,^2^ which combine information from different MR techniques.^2^ It is recommended as an annual screening tool for women with an increased risk of breast cancer.^3^, ^4^, ^5^ The backbone of breast MRI is dynamic contrast‐enhanced (DCE) MRI, which relies on intravenous injection of a gadolinium‐based contrast agent (GBCA) and enables assessment of lesion morphology as well as tracer kinetics, both important features for lesion characterization.^6^ However, an alternative technique that does not require GBCA injection is desirable because of growing concern related to the use of GBCAs,^7^ the additional staff costs because of the time required for intravenous injection,^8^ costs of the contrast agent itself,^8^ and patient discomfort.

In conventional DCE‐MR, both the wash‐in and wash‐out phase, minutes after contrast administration, are assessed. Studies on ultrafast DCE, with a temporal resolution of seconds in the intravenous contrast wash‐in phase, showed similar results in less time.^9^, ^10^ The early wash‐in phase is mainly determined by perfusion. Later on image contrast is dominated by leakage of the contrast agent into the extravascular space, due to increased vessel wall permeability in tumors.^11^

Arterial spin labeling (ASL) might be a promising noncontrast‐enhanced alternative for DCE‐MRI. Similar as ultrafast DCE‐MRI, ASL provides information on perfusion and vascularity, without being sensitive to vessel wall permeability. However, ASL does not require administration of contrast agent. ASL achieves perfusion contrast by labeling blood magnetically.^12^ In ASL, two images are acquired alternatingly; a label image, where blood is labeled magnetically, and a control image. The label and control images are subtracted to obtain an ASL image, where the static tissue signals cancel out and only labeled blood signal is left. Most common ASL techniques in brain and body, such as pseudo continuous ASL (pCASL),^13^, ^14^ and flow‐sensitive alternating inversion recovery (FAIR),^15^, ^16^ are spatially selective. In spatially selective techniques, labeling of blood takes place in the feeding arteries of the tissue of interest,^12^ resulting in a transit delay between the labeling location and arrival of labeled blood in the tissue. During the transit delay, labeled blood will not only flow from the labeling location into the tissue but will also decay with the T_1_ of blood, which is around 1.65 sec at 3 T.^14^ Thus for a delay of 1.65 sec, already 65% of ASL signal is lost. This poses a significant challenge for breast ASL. Blood flow in the internal mammary artery, feeding the breast is on the order of 19 cm/sec,^17^ which is lower than the blood flow in the carotid arteries feeding the brain, that is, 39 cm/sec.^18^ This complicates the use of spatially selective ASL techniques in breast.

Velocity‐selective ASL is an ASL‐technique that labels blood based on flow velocity instead of spatial location.^19^ All blood above a certain cutoff velocity gets labeled, so if the cutoff velocity is chosen low enough the transit delay is essentially eliminated. Hence, we hypothesized that VS‐ASL can be used in breast and has the potential to serve as a noncontrast‐enhanced perfusion method in breast cancer patients.

In this study, the technical feasibility of VS‐ASL in breast was investigated. Thus, the aim of this study was to investigate whether VS‐ASL generated enough contrast at a lesion location to identify increased perfusion and vascularity within breast lesions compared to surrounding tissue.

## Materials and Methods

### Subjects

This study was approved by the local ethics committee (METC Leiden Den Haag Delft NL70510.058.19) and informed consent was obtained from all participants. Patients were recruited via the outpatient clinic for breast cancer. Inclusion criteria were: scheduled for an MRI breast exam (either for screening or staging purposes), aged 18 years or over, and mentally competent. Exclusion criteria were: unable or unwilling to comply with breathing instructions, contra‐indications for gadolinium‐based contrast agents, and having previously undergone breast reduction treatment.

## Image Acquisition

All subjects were scanned in prone position on a 3 T Philips Ingenia Elition scanner (Philips, Best, The Netherlands), using a dedicated 16‐channel bilateral breast coil. For all scans, image‐based shimming (SmartBreast, Philips, Best, The Netherlands) was used to reduce B_0_‐related artifacts. A VS‐ASL and M_0_ scan were acquired prior to the standard clinical protocol. To reduce motion artifacts, the VS‐ASL and M_0_ scans were acquired with paced breathing; patients were asked to synchronize their breathing with image acquisition such that they held their breath (on exhalation) during the acquisition and then took shallow breaths in and out between acquisitions. Coaching was provided via the intercom, and patient cooperation was monitored using VitalEye (Philips, Best, The Netherlands).

The VS‐ASL scan was acquired with a single‐shot GE‐EPI readout.

Twenty transverse slices with 5 mm thickness, a 1 mm slice gap, an acquisition resolution of 2.75 × 2.75 mm^2^. The field‐of‐view was bilateral and was set to 200 × 119 × 196 mm^3^. The scan had a TE of 15 msec, and the TR was set to 6500 msec to produce a close‐to‐natural breathing rhythm. The fold‐over direction was right–left to prevent fold‐in signal of the heart. VS‐ASL labeling was performed using a pair of hyperbolic secant pulses and bipolar gradients, as described by Wong,^12^ using only a single VS‐ASL labeling module (i.e. excluding the second VS‐ASL labeling module).The VS‐ASL scans were acquired with a cutoff velocity of 2 cm/sec, velocity‐encoding direction feet‐head, VS‐ASL module duration of 50 msec, and hyperbolic secant pulses with a maximum B_1_ of 13.5 μT and a duration of 20 msec. The postlabel delay (PLD) was set to 1000 msec. Spectral presaturation with inversion recovery (SPIR)^20^ fat suppression was used, and background suppression pulses^14^ (hyperbolic secant), aimed to null fat signal, were applied 366 msec and 820 msec after labeling. Twenty‐one pairs of control and labeled images were acquired, resulting in a scan duration of 4 minutes 46 sec.

An M_0_ scan was acquired for calibration of the ASL signal. The M_0_ scan was acquired using the same readout as the VS‐ASL scan but with ASL labeling turned off. Four repetitions of the M_0_ image were acquired, resulting in a total scan time of 40 sec.

The clinical protocol included T_2_‐weighted turbo spin echo (TSE) Dixon, diffusion‐weighted imaging (DWI), DCE, and ultrafast DCE scans. The T_2_‐weighted Dixon was acquired with 83 slices, an acquisition resolution of 1.2 × 1.2 × 2.4 mm^3^, TE/TR of 140 msec/5856 msec, and a TSE‐factor of 24. DWI was acquired with b‐values of 500 s/mm^2^, 1000 s/mm^2^, and 1500 s/mm^2^, 42 slices, an acquisition resolution of 2 × 2 × 4 mm^3^, and a 2D SE‐EPI readout. Lastly, DCE and ultrafast DCE images were acquired with Dotarem (0.5 mmol/mL) as GBCA, using a dosage of 0.2 cc/kg. Ultrafast DCE was acquired with a 3D gradient‐echo T_1_‐weighted readout without fat suppression, using 136 slices and a spatial resolution of 1.3 × 1.3 × 1.3 mm^3^. DCE‐MRI was acquired using a 3D multishot turbo‐field echo (TFE) readout, using a TFE factor or 30, spectral attenuated inversion recovery (SPAIR)^21^ fat suppression, 140 slices, and a spatial resolution of 1.0 × 1.0 × 1.5 mm^3^. Before contrast injection, baseline DCE and ultrafast DCE‐images were acquired. Directly after contrast injection, the ultrafast DCE series of 14 time points with a temporal resolution of 3.9 s was started, and subsequently the regular DCE‐series of five time points with a temporal resolution of 1 minute 12 sec.

### Image Analysis

Image analysis was performed using MeVisLab (MeVis Medical Solutions AG, Fraunhofer MEVIS, Bremen, Germany). The breast VS‐ASL and M_0_ images were co‐registered to each other using a group‐wise image registration method.^22^ For each voxel *i*, the ASL subtraction signal ΔS_i_ was obtained by subtracting control (*C*_*r,i*_) and label (*L*_*r,i*_) images, and averaged over the total number of repetitions *R* (Eq. 1). An M_0_‐value was obtained by averaging over all *N* voxels within a manually drawn ROI_heart_ (researcher S.L.F in consensus with radiologist N.V.) in the left ventricle of the heart (Eq 2). The resulting perfusion‐weighted signal (PWS) value for each voxel *i* was obtained by dividing the ASL images with the M_0_‐value.

(1) $$∆S_{i}=\frac{1}{R}\;\sum_{r=1}^{R}\left(C_{r,i}-L_{r,i}\right)$$

(2) $$M_{0}=\frac{1}{N}\sum_{i_{\mathrm{ROI}-\text{heart}}=1}^{N}M_{0,i_{\mathrm{ROI}-\text{heart}}}$$

(3) $${\mathrm{PWS}}_{i}=\frac{∆S_{i}}{M_{0}}$$

Scans from the standard clinical protocol were assessed as part of the standard clinical protocol. In case a lesion was present, information regarding location, size, apparent diffusion coefficient (ADC) map reduction, and whether the lesion presents itself as mass (space‐occupying) or nonmass (areas of enhancement without clear space‐occupancy) according to the BI‐RADS lexicon^23^ was extracted from the clinical report. In addition, images from the clinical DCE, ultrafast DCE scans and the ADC maps calculated from the clinical DWI‐scans were obtained from the clinical patient database. Ultrafast DCE images at 10 sec after enhancement of internal thoracic artery, reflecting mainly perfusion, were obtained to compare to the VS‐ASL images. All patients included for staging purposes received a biopsy prior to MRI, while all patients included for screening purposes only received a biopsy post‐MRI in case there was an indication based on the MRI results. When available, lesion characterization from biopsy was obtained from the clinical reports.

Three radiologists (N.V./T.K./M.N.J.M.W.) with 3–25 years of experience in breast radiology scored the PWS‐maps of the VS‐ASL scans on lesion visibility and artifacts, based on a 3‐point Likert scale. For lesion visibility, a score of 1 was defined as: “signal at the location of the lesion can be distinguished from surrounding tissue,” 2: “suspicion that signal at the location of the lesion is deviant from surrounding tissue,” 3: “signal at the location of the lesion cannot be distinguished from surrounding tissue.” For the artifact score, a score of 1 was defined as: “no or few artifacts are visible, artifacts can be distinguished from blood signal,” 2: “artifacts are visible, most can be distinguished from blood signal,” and 3: “artifacts have similar intensity as blood signal, and obscure assessment of the image.”

In addition, ROIs were drawn at the location of the lesion (in all slices occupied by the lesion) by a researcher (S.L.F) in consensus with a radiologist (N.V.). The PWS values of all *J* voxels in the ROI were averaged, to obtain a measure for signal enhancement of the lesion (PWS_lesion_) on the VS‐ASL PWS images (see Eq. 4).

(4) $${\mathrm{PWS}}_{\text{lesion}}=\frac{1}{J}\sum_{i_{\mathrm{ROI}-\text{lesion}}=1}^{J}{\mathrm{PWS}}_{i_{\mathrm{ROI}-\text{lesion}}}$$

### Histopathology

Microscopic evaluation of the tissue was performed for two patients (patients 2 and 7). One of the two patients underwent preventive bilateral breast ablation, and the other patient underwent lumpectomy. Thin section histopathology slices were analyzed to evaluate vascularity around the lesions, to be able to check for a biological basis of the VS‐ASL signal observed in these two patients.

### Statistical Analysis

The intra‐class correlation coefficient (ICC) was calculated to assess inter‐observer agreement, using SPSS Statistics (IBM Corp, Armonk, NY, USA), using a significance level of 0.05. An ICC of >0.74 was considered excellent, 0.6–74 good, 0.4–0.59 fair, and <0.4 poor.

## Results

**FIGURE 1:**
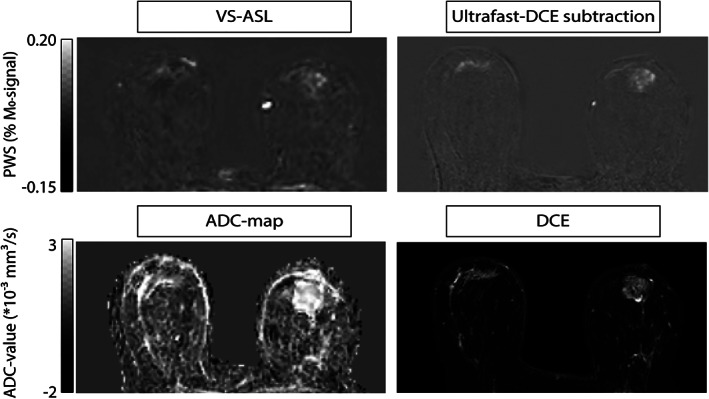
Representative patient with mass lesions; patient 5 with invasive lobular carcinoma grade 2 in right breast, and ductal carcinoma in situ grade 1 with adenosis in left breast. Signal in the VS‐ASL image corresponds with perfusion signal in early‐phase ultrafast DCE. Top row, from left to right: VS‐ASL, and the subtraction image of ultrafast DCE at a time point of 10 sec after contrast agent arrival in the internal thoracic artery. Bottom row, from left to right: ADC map calculated from b = 500–1000 s/mm^2^ and the first time point after contrast injection of the standard DCE scan, reflecting both perfusion and vessel wall permeability.

**FIGURE 2:**
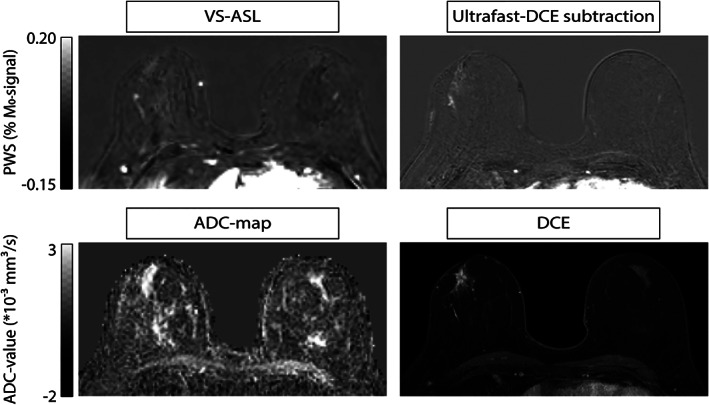
Representative patient with a non‐mass lesion; patient 9 with adenosis in the right breast. Signal in the VS‐ASL images corresponds with perfusion signal in the early phase ultrafast DCE. Top row, from left to right: VS‐ASL, and the subtraction image of ultrafast DCE at a time point of 10 sec after contrast agent arrival in the internal thoracic artery. Bottom row, from left to right: ADC map calculated from b 500–1000 sec/mm^2^, and the first time point approximately 1 minute after contrast injection of the standard DCE scan, reflecting both perfusion and vessel wall permeability.

Preliminary data comparing VS‐ASL with a SE‐EPI and GE‐EPI readout, and comparing multislice VS‐ASL with FAIR, a spatially selective technique, acquired with a single as well as a multislice readout is presented in Figs. 1, 2 and 3 in the Supplemental Material. Based on the standard clinical protocol, 7 out of 10 patients had a lesion on DCE‐MRI, of which one patient had bilateral lesions (Table 1). Four lesions were classified as invasive carcinoma, three as DCIS and one as a benign blunt duct adenosis. In one patient (patient 3), the lesion was not visible on DCE‐MRI, since it was a small focus of DCIS grade 1, which was likely completely removed by biopsy prior to the MRI. Of these eight lesions visible on DCE‐MRI, five presented as mass, with sizes ranging between 23 and 32 mm, and three as nonmass, with sizes ranging between 10 and 12 mm.

**TABLE 1:** Patient Characteristics and Results of Clinical MR Exam, Biopsy, and the Velocity‐Selective Arterial Spin Labeling (VS‐ASL) Scan

			Clinical MR Exam			Biopsy		VS‐ASL	VS‐ASL: Mean scores observers	
Patient	Left/Right Breast	Indication	Size (mm)	DCE‐Enhancement	ADC Reduction	Hormone Receptors	Lesion type	PWS at lesion (%M0)	Lesion visibility	Artifact
1	‐	Screening	‐	‐	‐	‐	‐	‐	‐	2
2	Left	Screening	11	Non‐mass	Yes	‐	DCIS 2	0.50	2	2
3	Left	Staging	6	Not visible^a^	No	‐	DCIS 1	‐	‐	2.6
4	‐	Screening	‐	‐	‐	‐	‐	‐	‐	2.3
5	Left	Staging	27	Mass	Yes	ER+, PR−, HER2‐ (right)	DCIS 1 + adenosis	0.70	1.3	2
	Right		32	Mass	Yes	‐	Invasive lobular carcinoma 2	0.32	1	1.3
6	Right	Staging	30	Mass	Yes	ER+, PR−, HER2‐	Invasive carcinoma NST 2	0.76	1	1
7	Left	Staging	23	Mass	Yes	ER+, PR+, HER2+	Invasive carcinoma NST 2	0.78	1	1
8	Right	Staging	12	Non‐mass	No	ER−, PR−, HER2‐	Invasive carcinoma NST 3	0.17	2.3	2.3
9	Right	Staging	10	Non‐mass	Yes	‐	Blunt duct adenosis	0.4	2	2
10	Left	Staging	33	Mass	Yes	ER+, PR−, HER2‐	Pleiomorphic lobular carcinoma	3.34	2	2.3

^a^ DCIS grade1 was found in a stereotactic biopsy specimen of 4 mm suspicious calcifications, in which probably the whole lesion was removed.

The results presented for the clinical MR exam and biopsy were taken from the clinical report.

DCE = dynamic contrast enhanced, ADC = apparent diffusion coefficient, ER± = estrogen receptor positive/negative, PR± = progresterone receptor positive/negative, HER2± = human epidermal growth factor receptor‐2 positive/negative, DCIS = ductal carcinoma in‐situ, PWS = perfusion‐weighted signal, Lesion visibility; 1 = “signal at the location of the lesion can be distinguished from surrounding tissue,” 2 = “suspicion that signal at the location of the lesion is deviant from surrounding tissue,” 3 = “signal at the location of the lesion cannot be distinguished from surrounding tissue.” Artifact score; 1 = “no or few artifacts are visible, artifacts can be distinguished from blood signal,” 2 = “artifacts are visible, most can be distinguished from blood signal,” 3 = “artifacts have similar intensity as blood signal, and obscure assessment of the image.”

There was an excellent inter‐observer agreement between the three readers for lesion visibility (ICC = 0.77, 95% confidence interval [CI] = 0.18–0.95) and artifact scoring (ICC = 0.84, IC = 0.55–0.95). Table 1 shows for all patients: the outcomes of the clinical MR exam, biopsy results, mean PWS of the lesion, and the lesion visibility and artifact scores averaged over the three observers. The mean lesion visibility score was 1.58. Mass lesions had a mean score of 1.27 and nonmass lesions 2.11.

Overall, VS‐ASL showed a comparable morphology to the early time point of ultrafast DCE. Larger arteries and veins were also clearly visualized. A representative patient with bilateral mass lesions is shown in Fig. 1, and a representative patient with nonmass lesions in Fig. 2.

**FIGURE 4:**
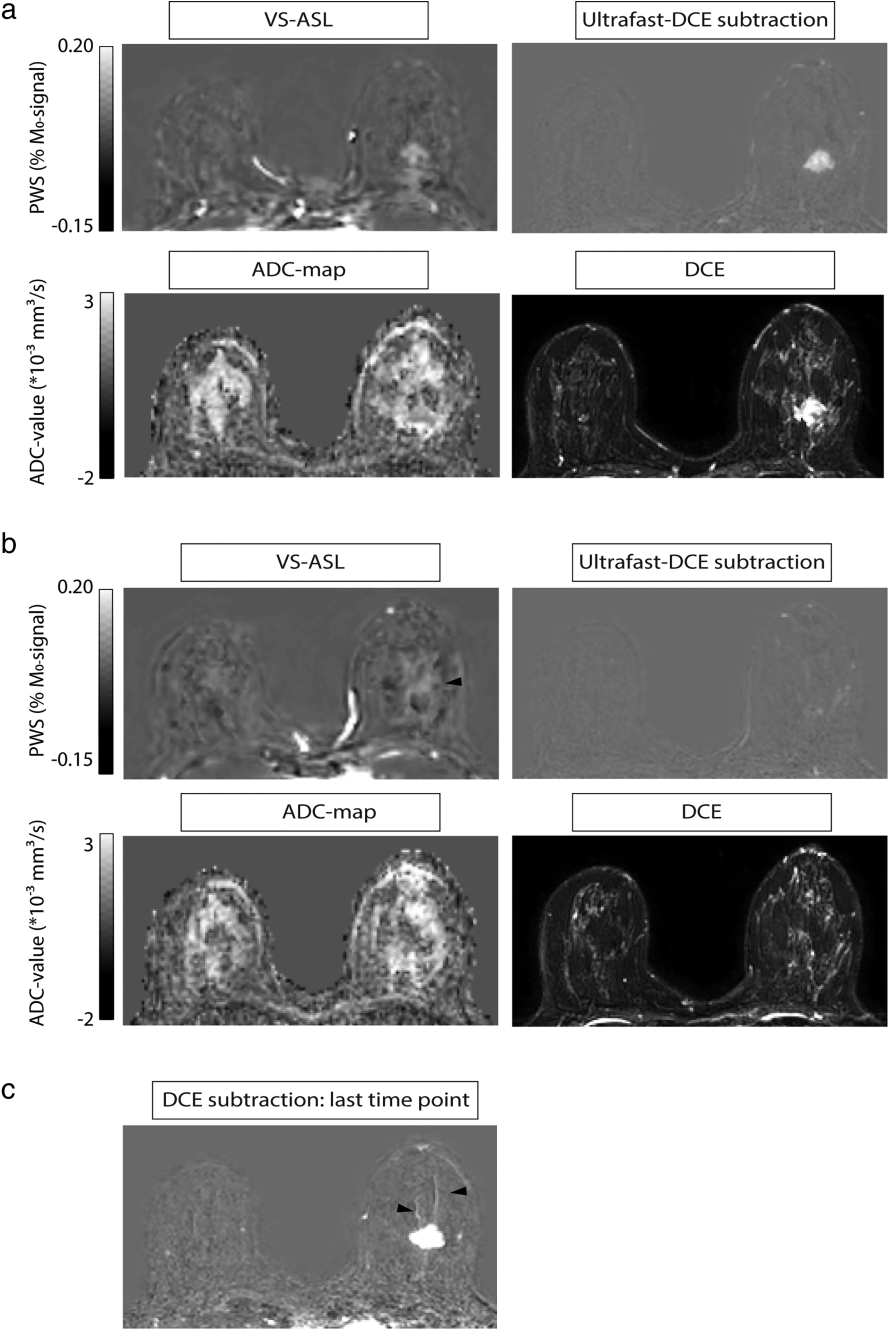
Representative patient with a segmental pattern of increased VS‐ASL signal; patient 7 with an invasive carcinoma grade 2 in the right breast. (a) slice containing the lesion, signal in the VS‐ASL image corresponds with perfusion signal in the early‐phase ultrafast DCE. (b) Segmental pattern of increased perfusion (indicated by black arrow) on the VS‐ASL images, in an adjacent slice to the tumor. There is no correlate of this pattern on the DCE, ultrafast DCE or the ADC map. However, in (c) notable vessels (indicated by black arrows) in the same area can be seen on the last time point of the DCE subtraction, likely showing a biological basis for the perfusion pattern seen in b. Histopathological data of this patient is shown in Supporting Information Fig. 4. Within sections A and B: Top row, from left to right: VS‐ASL, and the subtraction image of ultrafast DCE at a time point of 10 sec after contrast agent arrival in the internal thoracic artery. Bottom row, from left to right: ADC map calculated from b = 500–1000 sec/mm^2^, and the first time point after contrast injection of the standard DCE scan, reflecting both perfusion and vessel wall permeability.

**FIGURE 3:**
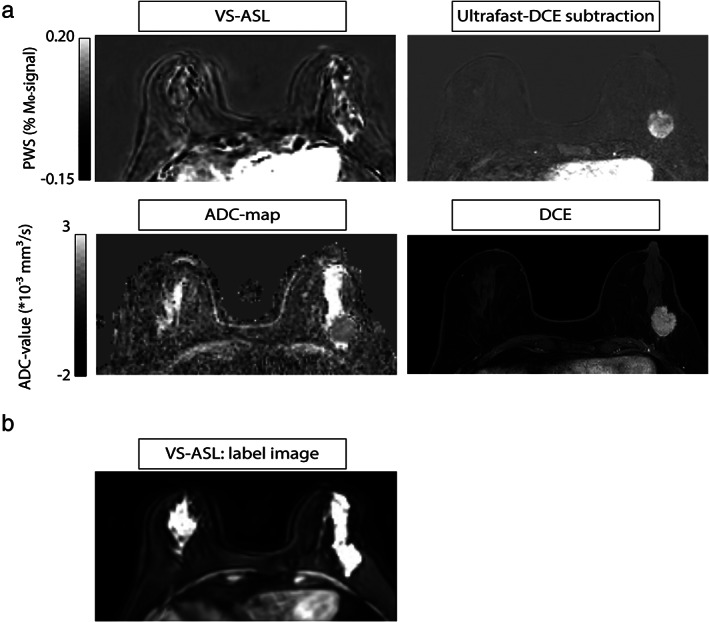
Representative patient with dense breast; (a) patient 10 with a pleiomorphic lobular carcinoma in the left breast, and dense breast. (b) The dense glandular tissue gives a relatively high signal contrast on the label images. Thus, motion can consequently easily lead to subtraction errors in the VS‐ASL image.

The mean artifact score was 1.9. Mass lesions had a mean artifact score of 1.53, and nonmass lesions 2.11. A representative patient with a high artifact score is shown in Fig. 3.

While scoring the data, an observation was made (N.V.) concerning increased VS‐ASL signal adjacent to the lesion, which was consistent with a segmental pattern. This segmental pattern of increased VS‐ASL signal was observed in three patients, see Fig. 4 for a representative case. No correlate was found on the ultrafast DCE images for these findings. However, on the DCE images, all three patients have notable vessels in these areas, see Fig. 4(c).

Histopathological analyses were performed for two patients: one diagnosed with a DCIS lesion (patient 2) and one with an invasive carcinoma (patient 7), see Table 1. In both patients, a clear difference in the size and hypertrophy of the small vessels surrounding the lesion, compared to the healthy breast tissue in the same patient was observed, see Fig. 4 in the Supplemental Material.

## Discussion

Our results demonstrated the feasibility of bilateral whole‐breast perfusion imaging using VS‐ASL in breast cancer patients. Moreover, a correspondence was observed in morphology of the patterns seen in VS‐ASL and early‐phase ultrafast DCE images.

Finding a noncontrast imaging technique to investigate tumor perfusion and vascularity is essential, as angiogenesis is one of the first signs of tumor growth and is critical for identifying breast cancers with a higher potential for invasion and metastasis.^24^, ^25^ Compared to traditional ASL methods, VS‐ASL addresses the challenging perfusion characteristics of the breast and enables bilateral whole‐breast perfusion imaging.

Previous studies investigating the feasibility of ASL in breast cancer employed spatially selective ASL techniques with a single slice planned on the tumor, using coronal slices to label the chest wall including the heart.^26^, ^27^, ^28^, ^29^ However, spatially selective ASL can lead to low SNR in case of multislice scanning in breast, due to slow flow. When using a multislice readout, the label is created further away from the breast tissue and the resulting prolonged transit delays, due to slow flow, will result in little label having reached the relevant slices at the time of acquisition. If the PLD is adjusted to for the prolonged transit delays, more SNR would be lost to T_1_‐decay of label, leading to poor image quality. Moreover, single‐slice FAIR would have, in addition to arterial signal, a contribution from venous signal flowing in posterior direction. Because whole‐breast coverage is a necessity for screening purposes, spatially selective ASL is unsuitable as a noncontrast screening method.

Overall, interobserver agreement of VS‐ASL was excellent for lesion visibility, and the artifact score. The mean lesion visibility was good, indicating that signal at the lesion could either be clearly distinguished from surrounding tissue or that there was a suspicion that the signal was deviant at the location of the lesion. The results suggest that in this limited sample size, nonmass lesions were less visible. However, the non‐mass lesions included in this study also had a smaller size. Visibility of the lesions on VS‐ASL corresponded well with visibility on the early phase ultrafast DCE images, confirming that VS‐ASL is a measure of perfusion and vascularity, similar to early phase ultrafast DCE.^11^ Parameters derived from ultrafast DCE‐MRI have been shown to be able to discriminate between benign and malignant lesions^30^ and have a strong relationship with tumor subtype.^31^ Thus, it would be interesting to investigate whether VS‐ASL could also provide sufficient information to discriminate between benign and malignant lesions.

While scoring the data, there was an unexpected finding by one of the observers: segmental patterns of increased perfusion were observed on the VS‐ASL PWS images adjacent to the lesion for three patients. In all cases, the pattern followed prominent vessels which were visible on DCE‐MR, suggesting a biological basis for the segmental perfusion patterns. The segmental perfusion patterns could indicate increased flow in the drainage area of the lesion due to a pathological vascular bed. Because vascularization has shown to be valuable in tumor diagnosis,^32^, ^33^ it would be interesting to investigate whether vascularization scores and observation of segmental perfusion patterns can aid detection of lesions in VS‐ASL images.

Scoring of the artifacts showed that artifacts were present in the VS‐ASL data, but that, overall, they could be distinguished from blood signal. In a few cases, the mean artifact score indicated that artifacts impacted proper assessment of the image. In all of these cases, the patient had extremely dense breast, according to the American College of Radiology classification, except for one patient who had a blood edema next to the lesion which obscured visibility. The artifacts observed in patients with dense breast are likely a result of a high contrast between fat and glandular tissue in the raw VS‐ASL images, which can easily lead to subtraction artifacts in case of small movements. Currently, two background suppression pulses were used. The short PLD of VS‐ASL prevents optimal suppression of both fat and glandular tissue. Possible strategies to improve the background suppression would be to increase the PLD and optimize timings for both tissue types, or to increase the number of background suppression pulses to three, to be able to suppress tissues across a broader range of T_1_ values.^34^ However, both strategies would come at the cost of SNR, by increased T_1_‐decay or by increased signal loss due to imperfect inversion pulses, respectively. Future studies are necessary to find out whether these proposed strategies result in a reduction of subtraction artifacts in patients with dense breast, at an acceptable SNR penalty.

Note that the VS‐ASL signal we measured is a combination of arterial and venous signal. Single VS‐ASL was used: meaning that only one VS‐ASL module was applied, instead of two as is commonly done in VS‐ASL.^19^ This second labeling module acts as a vascular crusher with a matched cutoff velocity, in both label and control condition. It has the effect of removing the venous and vascular contributions to the signal and defines the bolus duration, such that quantification of perfusion becomes possible. However, it comes at the expense of a reduction in SNR.^35^ This module was not applied by us, because being able to visualize venous and vascular signal could be clinically relevant in breast cancer patients, and, because we opted for maximum SNR for these first applications of VS‐ASL in breast, in view of the anticipated low ASL signal.

The single VS‐ASL sequence was played out with a cutoff velocity of 2 cm/sec, as has previously been done,^19^ while using a relatively short PLD. The choice of cutoff velocity is a trade‐off between labeling as close as possible to target tissue while preventing imaging artifacts caused by the increased gradient area. The motivation for using a short PLD was 2‐fold. First, the low cutoff velocity provides label already within the target region. Second, single VS‐ASL was used, that is, there is no second VS‐ASL labeling module. Conventionally in VS‐ASL, a longer PLD is required to ensure deceleration of the labeled blood before the second VS‐ASL module is applied. In our implementation, this was not the case, so a shorter PLD was used to prevent unnecessary loss of ASL‐signal due to T_1_‐decay.

Histopathologic analyses confirmed more vessels as well as more hypertrophic vessels at the border of a DCIS grade 2 and an invasive carcinoma, providing a possible biological basis for the VS‐ASL signal. Vascularity is an important characteristic of biologically more aggressive breast cancer subtypes.^32^, ^33^ Sensitive to vascularization and perfusion, VS‐ASL may also be sensitive to biologically aggressive breast cancer subtypes, similar as DCE‐MRI.^36^

### Limitations

First, as mentioned above, the background suppression should be optimized to reduce subtraction artifacts in patients with dense breast. Second, larger voxel sizes were used for VS‐ASL (2.75 × 2.75 ×  mm^3^) compared to DCE‐MRI (1 × 1 × 1.5 mm^3^) and ultrafast DCE (1.3 × 1.3 × 3 mm^3^), and since the desired detection limit of DCE‐MRI is 5 mm,^6^ future studies are needed to determine whether that detection limit can be met by VS‐ASL.

Third, in contrast to DCE‐MRI, no information on kinetics and vessel wall permeability is acquired with VS‐ASL. These parameters are currently used to differentiate between lesion subtypes; however, studies using ultrafast DCE‐MRI have shown that similar diagnostic accuracy can be achieved by only looking at the early perfusion phase, which does not include information on vessel wall permeability.^30^, ^31^ Future studies are necessary to investigate whether VS‐ASL could provide similar information as ultrafast DCE and whether other characteristics in VS‐ASL images could provide the same, or at least sufficient, discriminatory capability. Fourth, the VS‐ASL technique we used may not be the optimal velocity‐selective technique. For example, symmetric BIR‐8 VS‐ASL would be an interesting approach to limit erroneous labeling due to eddy currents and achieve higher robustness to B_0_ and B_1._ ^37^ Also velocity‐selective prepared inversion (VSI‐) ASL, which has demonstrated similar SNR as pCASL,^38^, ^39^ would be an interesting option. An adaptation to the original VSI‐ASL sequence has been published to improve the robustness for B_0_ and B_1,_ ^40^ which could prove essential for breast applications. Finally, the aim of this study was to do a technical proof‐of‐concept, so a small sample group was included. Future studies should include larger patient groups to establish the sensitivity and specificity of this technique, in terms of specific populations as well as lesion size and cancer subtype.

## Conclusion

Our results demonstrate the feasibility of VS‐ASL as a noncontrast‐enhanced measurement of perfusion and vascularity in breast. VS‐ASL is a promising technique that could add to a noncontrast mpMRI protocol for breast. A noncontrast mpMRI protocol would considerably reduce both patient discomfort and cost and could possibly be used as a prescreening method so that only patients with suspicious findings are referred for a DCE‐MRI exam.

## Supporting information


**Supporting Information Figure 1** Comparison of single‐shot multi‐slice GE‐EPI and SE‐EPI readout for VS‐ASL in a healthy volunteer. Top row: the VS‐ASL label images, before subtraction (raw image). Second row: the VS‐ASL subtraction images. Third row: the corresponding T_2_‐weighted image. The vascular signal in VS‐ASL (black arrows), which corresponds to the vascular signal in the T_2_‐weighted image (white arrows), is more clearly visible with GE‐EPI readout compared to SE‐EPI readout. Therefore, a GE‐EPI readout was used for the patient scans. Note, that the ASL images in healthy volunteers mainly show vascular signal, because the perfusion levels of healthy tissue are too low to measure. Scan parameters for both sequences were the same as described in the main document, except with a TE of 25 ms and half‐scan factor of 0.7 for SE‐EPI.
**Supporting Information** Figure 2. Slice planning for A) VS‐ASL, B) multi‐slice FAIR and C) single‐slice FAIR. The orange box represents the imaging stack, and the green box represents the area where label is created. As can be seen in A) label is created also inside the imaging region when using VS‐ASL labeling, while this is not the case for FAIR. With multi‐slice FAIR the labeling takes place further from the imaging region (B), leading to a reduced SNR in the resulting ASL image. In single‐slice FAIR the labeling takes place close to the imaging region (C), and label is created on both sides of the imaging slice, so more signal can be expected. However, single‐slice imaging is not suitable for screening purposes, making FAIR unsuitable for breast screening purposes.
**Supporting Information** Figure 3. Comparison of multi‐slice VS‐ASL and single‐slice/multi‐slice FAIR. Multi‐slice VS‐ASL and single‐slice FAIR both show vascular signal (black arrows) that corresponds to vascular signal on the T_2_‐weighted scan (white arrows), while multi‐slice FAIR does not. FAIR labeling takes place outside of the field‐of‐view, and with multi‐slice scanning this means transit delays increase. Firstly, blood flow in breast in relatively slow, so not enough ASL‐signal has arrived in the region of interest yet at the time of acquisition. Secondly, label decays with T_1_, so it would also not be an option to match the PLD to the transit delay. Because then most ASL‐signal will likely have decayed already. In addition, in contrast to single‐slice FAIR there is no contribution of venous signal in multi‐slice FAIR. VS‐ASL labels based on velocity, non‐spatially‐selective, also already in the region of interest. So in case of VS‐ASL there is no transit delay, making it compatible with a multi‐slice readout in breast. Acquisition parameters VS‐ASL: the same settings were used as described in the main document; however, the image orientation was changed from transverse to coronal to match the FAIR acquisition. Acquisition parameters FAIR: FAIR used a frequency offset corrected inversion (FOCI) pulses. The selective inversion slab was aligned with the imaging stack, with an additional width of 6 mm on both sides, and inversion time was set to 1200 ms. Single‐slice FAIR was acquired using a single slice of 8 mm to achieve the shortest transit delay [27, 29]. Multi‐slice FAIR uses the same imaging stack as used for VS‐ASL (20 slices with 5 mm slice thickness and 1 mm slice gap) to achieve full breast coverage as required for screening. Acquisition parameters T2‐weighted scan: T_2_‐weighted turbo spin echo (TSE) Dixon, 47 slices, acquisition resolution of 1.2 x 1.2 x 2.4 mm^3^, field of view of 300 x 360 x 123 mm^3^, TE/TR of 140 ms / 5850 ms.
**Supporting Information** Figure 4. Histopathology results in two patients. A) Increased number of vessels and increased number of hypertrophic vessels (indicated by black arrows) are observed with a CD 31 vessel wall staining in patient 2 with an 11 mm DCIS grade 2 lesion. B) These vessels are not visible in a similar location in the contralateral healthy breast. C) Hypertrophic vessels (indicated by black arrows) at the border of the 22 mm invasive carcinoma grade 2 in patient 7, observed with a HE staining. D) These vessels are not visible in a healthy tissue of the same patient. E) CD 31 vessel wall stained section confirming the higher number of vessels at the location of the lesion.
