# Supplementary material for: Feasibility of Velocity‐Selective Arterial Spin Labeling in Breast Cancer Patients for Noncontrast‐Enhanced Perfusion Imaging
Source: J Magn Reson Imaging. 2021 Jun 13;54(4):1282–91. doi: 10.1002/jmri.27781 (PMC8518819; doi:10.1002/jmri.27781)
Supplement: Supplementary file 1 — Supporting Information Figure 1 Comparison of single‐shot multi‐slice GE‐EPI and SE‐EPI readout for VS‐ASL in a healthy volunteer. Top row: the VS‐ASL label images, before subtraction (raw image). Second row: the VS‐ASL subtraction images. Third row: the corresponding T2‐weighted image. The vascular signal in VS‐ASL (black arrows), which corresponds to the vascular signal in the T2‐weighted image (white arrows), is more clearly visible with GE‐EPI readout compared to SE‐EPI readout. Therefore, a GE‐EPI readout was used for the patient scans. Note, that the ASL images in healthy volunteers mainly show vascular signal, because the perfusion levels of healthy tissue are too low to measure. Scan parameters for both sequences were the same as described in the main document, except with a TE of 25 ms and half‐scan factor of 0.7 for SE‐EPI. Supporting Information Figure 2. Slice planning for A) VS‐ASL, B) multi‐slice FAIR and C) single‐slice FAIR. The orange box represents the imaging stack, and the green box represents the area where label is created. As can be seen in A) label is created also inside the imaging region when using VS‐ASL labeling, while this is not the case for FAIR. With multi‐slice FAIR the labeling takes place further from the imaging region (B), leading to a reduced SNR in the resulting ASL image. In single‐slice FAIR the labeling takes place close to the imaging region (C), and label is created on both sides of the imaging slice, so more signal can be expected. However, single‐slice imaging is not suitable for screening purposes, making FAIR unsuitable for breast screening purposes. Supporting Information Figure 3. Comparison of multi‐slice VS‐ASL and single‐slice/multi‐slice FAIR. Multi‐slice VS‐ASL and single‐slice FAIR both show vascular signal (black arrows) that corresponds to vascular signal on the T2‐weighted scan (white arrows), while multi‐slice FAIR does not. FAIR labeling takes place outside of the field‐of‐view, and with multi‐slice scanni [file JMRI-54-1282-s001.docx]

**Supporting Information Figures**


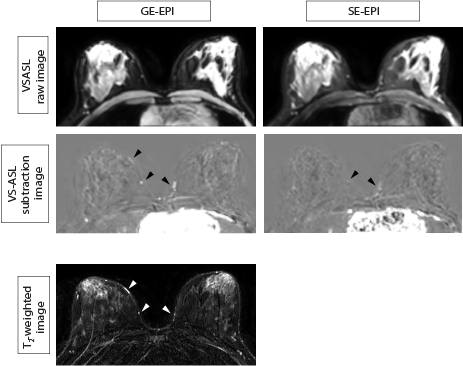


Supporting Information Figure 1. Comparison of single-shot multi-slice GE-EPI and SE-EPI readout for VS-ASL in a healthy volunteer. Top row: the VS-ASL label images, before subtraction (raw image). Second row: the VS-ASL subtraction images. Third row: the corresponding T_2_-weighted image. The vascular signal in VS-ASL (black arrows), which corresponds to the vascular signal in the T_2_-weighted image (white arrows), is more clearly visible with GE-EPI readout compared to SE-EPI readout. Therefore, a GE-EPI readout was used for patient scans. Note, that the ASL images in healthy volunteers mainly show vascular signal, because the perfusion levels of healthy tissue are too low to measure. Scan parameters for both sequences were the same as described in the main document, except that for the SE-EPI scan the TE was set to 25 ms and a half scan factor was set to 0.7.


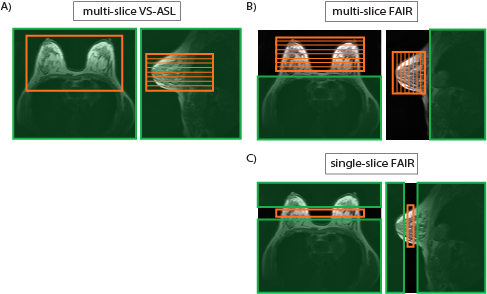


Supporting Information Figure 2. Slice planning for A) VS-ASL, B) multi-slice FAIR and C) single-slice FAIR. The orange box represents the imaging stack, and the green box represents the area where label is created. As can be seen in A) label is created also inside the imaging region when using VS-ASL labeling, while this is not the case for FAIR. With multi-slice FAIR the labeling takes place further from the imaging region (B), leading to a reduced SNR in the resulting ASL image. In single-slice FAIR the labeling takes place close to the imaging region (C), and label is created on both sides of the imaging slice, so more signal can be expected. However, single-slice imaging is not suitable for screening purposes, making FAIR unsuitable for breast screening purposes.


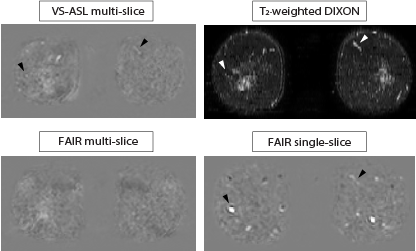


Supporting Information Figure 3. Comparison of multi-slice VS-ASL and single-slice/multi-slice FAIR. Multi-slice VS-ASL and single-slice FAIR both show vascular signal (black arrows) that corresponds to vascular signal on the T_2_-weighted scan (white arrows), while multi-slice FAIR does not. FAIR labeling takes place outside of the field-of-view, and with multi-slice scanning this means transit delays increase. Firstly, blood flow in breast in relatively slow, so not enough ASL-signal has arrived in the region of interest yet at the time of acquisition. Secondly, label decays with T_1_, so it would also not be an option to match the PLD to the transit delay. Because then most ASL-signal will likely have decayed already. In addition, in contrast to single-slice FAIR there is no contribution of venous signal in multi-slice FAIR. VS-ASL labels based on velocity, non-spatially-selective, also already in the region of interest. So in case of VS-ASL there is no transit delay, making it compatible with a multi-slice readout in breast. Acquisition parameters VS-ASL: the same settings were used as described in the main document; however, the image orientation was changed from transverse to coronal to match the FAIR acquisition. Acquisition parameters FAIR: FAIR used a frequency offset corrected inversion (FOCI) pulses. The selective inversion slab was aligned with the imaging stack, with an additional width of 6 mm on both sides, and inversion time was set to 1200 ms. Single-slice FAIR was acquired using a single slice of 8 mm to achieve the shortest transit delay. Multi-slice FAIR uses the same imaging stack as used for VS-ASL (20 slices with 5 mm slice thickness and 1 mm slice gap) to achieve full breast coverage as required for screening. Acquisition parameters T2-weighted scan: T_2_-weighted turbo spin echo (TSE) Dixon, 47 slices, acquisition resolution of 1.2 x 1.2 x 2.4 mm^3^, field of view of 300 x 360 x 123 mm^3^, TE/TR of 140 ms / 5850 ms.


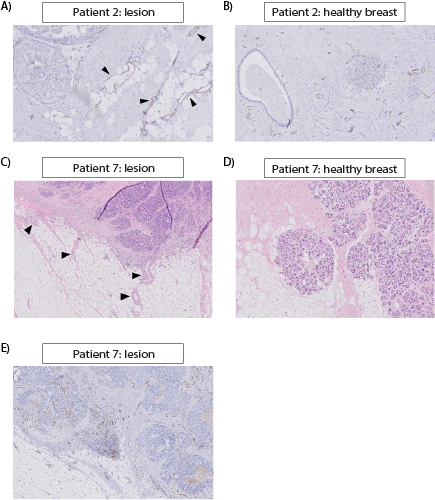


Supporting Information Figure 4. Histopathology results in two patients. A) Increased number of vessels and increased number of hypertrophic vessels (indicated by black arrows) are observed with a CD 31 vessel wall staining in patient 2 with an 11mm DCIS grade 2 lesion. B) These vessels are not visible in a similar location in the contralateral healthy breast. C) Hypertrophic vessels (indicated by black arrows) at the border of the 22mm invasive carcinoma grade 2 in patient 7, observed with a HE staining. D) These vessels are not visible in a healthy tissue of the same patient. E) CD 31 vessel wall stained section confirming the higher number of vessels at the location of the lesion.
